# Supplementary material for: SID-1 Domains Important for dsRNA Import in Caenorhabditis elegans
Source: G3 (Bethesda). 2017 Oct 12;7(12):3887–99. doi: 10.1534/g3.117.300308 (PMC5714486; doi:10.1534/g3.117.300308)
Supplement: Supplementary file 1 [file 3887FigureS1.pdf]

```
CBR-CHUP-1      ---MKALQAIFILIFSELI------------------STQKPEVIPAKWDVLYE--KETE
CBN-CHUP-1      ---MKKLSIIFILIFFQIV------------------SSQVPEVIPAKWDVLYQ--KETG
CRE-CHUP-1      ---MKATQCIFLLIFLEVV------------------KSQLPQVIPAKWDVMYG--KETG
CEL-CHUP-1      ---MRTSQAIFILIFLDSV------------------RNQSPQVIPAKWDVVYE--KETG
CJP-SID-1       -----MYNEIILRVVRAVLGRLADPNVTQYAS-----NNEPESIIIKHLNKKLT--NETL
CEL-SID-1       -----MIRVYLIILMHLVIGLTQNNSTTPSPII----TSSNSSVLVFEISSKMKMIEKKL
CBN-SID-1       MKGSGSILGCLLIVLLGQLGLSDNITDKEPSELDFTKNNETGPIIVRQLHSKTKMFNATL
CRE-SID-1       MVGSRFFASCLLLVLLGKLNCSGNNTDNGVQE-----TDEENPVITREFPSKKKVYETTL
CBR-SID-1       MIGSRYFVSGVLLLLLGRFGLSEQD------------NNETSPISVREFPARRRQINDTL
                          .: :.   .                    .   :            : .
                     M1I

CBR-CHUP-1      KNMSLTIFRFNVEKQY--SVARIIMSCNES----TEHNPLLAV-FREKVAILSLQVPLIV
CBN-CHUP-1      KNMSMTIFRFRVEEQY--SVARIIMSCNES----TEHNPLLAV-FREKLAILSLQVPLIV
CRE-CHUP-1      KNMSLTIFRFKVEEKY--SVARIIMSCNES----TEHNPLLAV-FREKLAVLSLQVPLIV
CEL-CHUP-1      HNMSLTVFRFQVKEQY--SVARIIMSCNES----TEHNPLLAV-FREKLAILSLQVPLIV
CJP-SID-1       KAHTVNVLYNSLYTQSILDLTRVSVTIDEPWKFDTVHGTILEATVSNGRDNFLLKLPVVY
CEL-SID-1       EANTVHVLRLELDQSFILDLTKVAAEIVDSSKYSKEDGVILEVTVSNGRDSFLLKLPTVY
CBN-SID-1       KPGKVHLYYLSLDDSYVLDLMRIAAEVIDPVLYKNEKDAILEVTVSNGRDNFNLKLPVIY
CRE-SID-1       KPNAVHVYYLKMNETYILDLIRVAAEITHPTLFEKEEDTILDVTVSNGRDNFVIKLPVVY
CBR-SID-1       VANTVHVYYLKLDETFILDLTRISADVSEPALFRKEVDTILEVTVSNGRDNFILKLPMIY
                    : :    :      .: .:      .    .  . :* . . :    : :::* :
                                                                        P108S

CBR-CHUP-1      DN---YEYSQVARTLCP--FTEYKEGEA---------------FTVEVTSSRPVHYNFRA
CBN-CHUP-1      DN---YEYSQVARTLCP--FTEYKEGEA---------------FTVEVTSSRPVHYNFRA
CRE-CHUP-1      DN---YEYSQVARTLCP--FTEYKEGEA---------------FTVEVTSAQPVRYNFRA
CEL-CHUP-1      DN---YEYSQVARTLCP--FTEYKEGEA---------------FTVEVTSSRPVHYNFRA
CJP-SID-1       KNVTLYSDGKVLNPLLPEDFGDRKLRKNKQRFLPDNDENYHQNLIITVHSTLHEDIKYSV
CEL-SID-1       PNLKLYTDGKLLNPLVEQDFGAHRKRHR------IGDPHFHQNLIVTVQSRLNADIDYRL
CBN-SID-1       PNLTLYSYGKLLNPLISDDFGPKISKKR-------RNSTGNQTLVISVQSRLKVDLDYRL
CRE-SID-1       PEGSLYTYGKLLNPLIPDDFGPKRSKKN------TENSTGTQNLIVTVQSRLRVDIDYKL
CBR-SID-1       PDLTLYSYGKLLNPLRKEDFGPKRSKKN----IPSENSTLSQNLVITVQSRLRVDIDYQL
                 :   *  .:: ..*    *                       : : * *      .:
                                  D130N

CBR-CHUP-1      ELVH------NFYLYNDSKRLVTASASEPVYLR------YDIPNGVDSVAVHVLSNTTTC
CBN-CHUP-1      ELVQ------NFHLYNDAKRLVTASASEPVYLR------YDIPGDVDSVAVHVDSNSSIC
CRE-CHUP-1      ELVK------NFFLYNNGKRLVTESASEPVYLR------YDIPDDVDSVAVHVDSNSTTC
CEL-CHUP-1      ELVQ------NFYLYNNSQRLVTASASEPVYLR------YDIPGDVDSVAVHLDSNSTIC
CJP-SID-1       SVTHLDRSQYY-VKFKPGETRINQNDQHLTVVKPLGYFLDAKEQNVNAFHITVTSDDDIC
CEL-SID-1       HVTHLDRAQYDFLKFKTGQTTKTLSNQKLTFVKPIGFFLNCSEQNISQFHVTLYSEDDIC
CBN-SID-1       HLTRLDRSQYN-LKFKPGQSTKTLSNQKLTFVKPIGFFLDAIEQNVKSFHITLLSDDDFC
CRE-SID-1       FLTHLDRSQYD-LKFKPGQSEKTLQNQKLTFVKPMGFFLDAKEQDVKSFHITVTSEDDIC
CBR-SID-1       HLTRLDRSQYN-LSFKEGQSTKTLNNQKLTFVKPIGFFLDTEEQIVKSFHITLTSADDIC
                 :..          :: .:   . . .  . :.            :. . : : *    *
                      R172W                         G201R
                       A173T                        G201E
                                                  P199L

CBR-CHUP-1      MTVSVQKIGCPVFDLPDNVNSMGLHQTMTTSATIPV-----EKSRMSSFYVIFVVNTNDD
CBN-CHUP-1      MTVSVQKIGCPIFDLPNNVNSMGLHQTMTTSATIPV-----EKSRMSSFYVVFVVNTNDD
CRE-CHUP-1      MTVSVQKIGCPVFDLPDNVNSMGLHQTMTTSATIPV-----EKSRMSNFYVVFVVNTNDD
CEL-CHUP-1      MTVSVQKIGCPVFDLPDNVNSMGLHQTMTTSATIPV-----EKSRMSSFYVVFVVNTNDD
CJP-SID-1       ANLITVPADEPLYDRRVDLDKVDNRRILSFTRRADVLFPNSEIANFEKFRIFVLISPDDS
CEL-SID-1       ANLITVPANESIYDRSVISDKTHNRRVLSFTKRADIFFTETEISMFKSFRIFVFIAPDDS
CBN-SID-1       ASLITVPANESIYDRPVDADKADNNRVITFTKRADIFFSQTEIETFKSFRIFVFISPVDS
CRE-SID-1       ANVITVPANESIYDRPVDSDKADNQRVITFNRRADIFFSEKEIELFKSFRIFVFVSPVDS
CBR-SID-1       ANVITAPANESIYERPVDSDRADNRRVLTFTRRADIFFSETEIQLFKTFRIFVFISPVNA
                 .: .   . .:::     :    .. :: .    :     *   :..* :...: . :
```

```
CBR-CHUP-1      LCSEILSIKPNRPTKFPLRLKSFNVTIE-SSMKIFDYIIPIVFWACILLLVTLVVFVYHY
CBN-CHUP-1      LCSEISSIKPIRPTKFPLRLKSFNVTIE-SSMKTSDYTIPIVFWACILFLVTIVVFVYHY
CRE-CHUP-1      LCSEIMSIKPN----------PFNVTIE-SSMKIFDYTIPIVFWACVLLLVTIVVFVYHY
CEL-CHUP-1      LCSEILSIKPNKPTKFPLRMKSFNVTIE-SSMKIFDYTIPIVFWACILLLVTIVVFVYHY
CJP-SID-1       LC-GASSRKTALQNK--------KLSLEFVKLNDASYSVPLIA-MFTFLAIPALIFLP--
CEL-SID-1       GCSTNTSRKSFNEKK--------KISFEFKKLENQSYAVPTAL-MMIFLTTPCLLFLPIV
CBN-SID-1       PCSTNSSRKTFNENK--------KVTFEFAKLEPSSYFFPTAL-MMAFLATPVLLFVPAL
CRE-SID-1       PCSLKTSRKTFNEQK--------KMTFEFKKLEPDSYFMPTLA-MIAFFISPCLIVASIL
CBR-SID-1       PCSGNTSRKNYNEIK--------KITFDFTRLEPNSYFVPTLA-MLVFFASPCLIFIASL
                 *    * *              :::::   ::  .* .*       ::  . ::.
                                                                   P328L

CBR-CHUP-1      FDGIWERRFLSRAYTHLEDNAQDERLRDFYEFKRMSEDDDLRDY----DILTDCKDMM--
CBN-CHUP-1      FDGIWERRFISRAYTHLEDNAQEERIRDFYDFQRMSEDEDLKDY----DLLLDCKDMM--
CRE-CHUP-1      FDGIWERRFLSRAYTHLEDDAQEERIRDFYDFKRMSEDDDLKDY----DLLTDCKDMM--
CEL-CHUP-1      FDGIWERRFVSRAYTHLEDNAQEQRIRDFYDFQRMSEDDDLKDY----DLLTDCQDMM--
CJP-SID-1       FVTLTRFRSMPIATDHLLDLST--------DLSSSSQPSPLIVL----DESATDNSLG--
CEL-SID-1       INIIKNSRKLAPSQSNLISFSPVPSEQRDMDLSHDEQQNTSSELENNGEIPAAENQIVEE
CBN-SID-1       ILN--RIRNTSTSQSTLI------------SFAPDTPDQSYQVE----EGPSTDNDI---
CRE-SID-1       AVNVWRNRDPSNTSADLV------------SFESDEPNHP--------NANTSDEQLV--
CBR-SID-1       TVNVIRNR--SDLVADLI------------SFSSDQSANTSAIA----ENNMAHNEIA--
                       *  .     *             .:                :     :.:

CBR-CHUP-1      VVRAKT-TLTVAD-----LSMTPY--ETREMQYDVYKIALAIIGIFYNITVLQLIISKAG
CBN-CHUP-1      VVRAKA-TLTVAD-----LSMTPY--ETREQQYDVYKIALAIIGIFYNITVLQLIISKAG
CRE-CHUP-1      VVRAKA-SLTVAD-----LSMTPY--ELREQKYDVYKIALAIIGIFYNITVLQLIISKAG
CEL-CHUP-1      VVRAKA-SLTVAD-----LSMTPY--EERELKYDVYKIALAIIGIFYNITVLQLIISKAG
CJP-SID-1       IAEVNA-DITVLED----VKLT----DSLSLHGEMLRYPVAIILPVLMHTAVEYHNWSKS
CEL-SID-1       ITAENQ-ETSVEEGNREIQVKIPLKQDSLSLHGQMLQYPVAIILPVLMHTAIEFHKWTTS
CBN-SID-1       VVPEEE-NLQNQE-----GNIIPLPEDSLSLHGQMLKYPLAIILPVFMHTAVEYHEWTTS
CRE-SID-1       VMEEEEVNLQNHENLQNHVEAVSVKQDSLSLHGQVLRYPIAIILPVLMHTAVQFHNFTFS
CBR-SID-1       VIPEEE-NLQVQE-----IEPIPIKQDSLSLHGQMFKYPVALILPVLMHTGVEFHNFTTS
                :   :       :             :  . : :: . .:*:*  .   * ::    . .

CBR-CHUP-1      NLRQSGDLDECTFNFQCARPLWYFVAFNNVVSNCGYVYFGSLIIVMNYCRERSFRRLFAV
CBN-CHUP-1      SLRQSGDLDECTFNFQCARPLWYFVAFNNVVSNGGYVYFGSLIIVMNYCRERSFRRLFAV
CRE-CHUP-1      SLRQSGDLDECTFNFQCARPLWYFVAFNNVVSNGGYVYFGLLIIVMNYCRERSFRRLFAV
CEL-CHUP-1      SLRQSGDLDECTFNFQCARPLWYFVAFNNVVSNGGYVYFGTLIIVMNYCRERSFRRLFAV
CJP-SID-1       SMANRDEM--CFYNHACANPYGEFRAWNNIISNIGYAIYGLAFVAITVGRRRRSRQLVG-
CEL-SID-1       TMANRDEM--CFHNHACARPLGELRAWNNIITNIGYTLYGAIFIVLSICRRGRHEYSHV-
CBN-SID-1       TMANRDEM--CFHNNACARPYGELRAWNNIISNIGYAIYGLVFIAITMCRRWRHGSPVV-
CRE-SID-1       TMANRDEM--CFHNSACSKPLGELRAWNNMISNIGYAIYGFVFILITMFRRWRHHSALV-
CBR-SID-1       TMANRDEM--CFHNNACAKPLGELRSWNNMISNIGYAIYGLVFIMVTMCRRWRHHSPLV-
                .: : .::  * .*  *:.*   : ::**:::* **. :*  :: :.  *
                          C464Y                   G488E
                                                       G493R

CBR-CHUP-1      QPTLAERYGLPQHSGLMTAIGLAVIMEGISSATYHVCPNNINYQFDTALMYVIGMLGKLK
CBN-CHUP-1      QPTLAERYGLPQHSGLMTAIGLAVIMEGISSATYHVCPNNINYQFDTALMYVIGMLGKLK
CRE-CHUP-1      QPALAERYGLPQHSGLMTAIGLAVIMEGISSATYHVCPNNINYQFDTALMYVIGMLGKLK
CEL-CHUP-1      QPTLAERYGLPQHSGLMTAIGLAVIMEGISSATYHVCPNNINYQFDTALMYVIGMLGKLK
CJP-SID-1       -------YGVHESTLIDVTIGVFMVLQAIASATYHICPSNVSFQFDTPCIQMICCLLIIR
CEL-SID-1       -------FGTYECTLLDVTIGVFMVLQSIASATYHICPSDVAFQFDTPCIQVICGLLMVR
CBN-SID-1       --------GIYECTLLDVTIGVFMILQAIASATYHICPSDVAFQFDTPCIQVICGLLIIR
CRE-SID-1       --------GTYECTLLDITIGVFMILQAIASATYHICPSDIAFQFDTPCIQVICGLLIIR
CBR-SID-1       --------GTYECTLLDITIGLFMILQAIASATYHICPSDIAFQFDTPCIQVICGLLIIR
                        *  : : :  :**: ::::.*:*****:**.:: :****. : :*  *  :.
                                            I534N                    C559Y
                                              S536I                        R565C
```

```
CBR-CHUP-1      IWSLRHPDMVVSAYHAFGFLGIFLMAAIAGVYVHNMIFWIMFSIIYIGSMMLISLEFYFK
CBN-CHUP-1      IWSLRHPDMVVSAYHAFGFLGMFLMAAIAGVYVHNMIFWIMFSTIYIASMMLVSLEFYFK
CRE-CHUP-1      IWSLRHPDMVVSAYHAFGFLGIFLMAAIAGVYVHNMIFWIMFSIIYIGSMMLISLEFYFK
CEL-CHUP-1      IWSLRHPDMVVSAYHAFGFLGVFLMAAIAGVYVHNMIFWALFSIIYIASMLLVSLEFYFK
CJP-SID-1       QWLVRL-ESPSAAYSNLLLVVVVSVNFLVSALSKTWYIRYLIAGIHMFIISSICL-----
CEL-SID-1       QWFVRH-ESPSPAYTNILLVGVVSLNFLISAFSKTSYVRFIIAVIHVIVVGSICL-----
CBN-SID-1       QWRVRQ-ESPSASYTNLLLFGVVSLNFLISAFSKAKYVRYLIAMIHISAIASMCL-----
CRE-SID-1       QWLVRK-ESPSPAYTNMLLLAVVSLNFLISTLSKSSRVRYVIATIHFIVVATICL-----
CBR-SID-1       QWLVRQ-ESPSPAYTNILLFCVVSLNFLISACSKASGIRYLIAIIHFGVVATVCL-----
                 * :*  :   .:*  : :. :. :  : ..  :   .. ::: *:.  :  :.*
                    R570C

CBR-CHUP-1      GIWTLNIRELRNSIRMSWATSRHLSC--IVPAYKARFFVILLLNMANTAVVVYGLDAHPK
CBN-CHUP-1      GIWTLNLRELRNSVRMAWASSKRLSC--VIPAYKARFFVILLLNLANTAVVVYGLEAHPK
CRE-CHUP-1      GIWTLNIRELRNSVRMAWASSRRLSC--IMPAYKARFFVILMLNIVNTAVVVYGLEAHPK
CEL-CHUP-1      GIWTLNLRELRNSIRLSWVSSRHLSC--VVPAYKARFFVILLLNIANTAVVVYGLEAHPK
CJP-SID-1       --------AKKRMLGINESAFRKQISLTFIIYYSICNITMLLVYLVSAKI-------HLN
CEL-SID-1       --------AKERSLGSEKLKTRF-----FIMAFSMGNFAAIVMYLTLSAF-------HLN
CBN-SID-1       --------AKRRTLETKKTFQV------FTACFAAINFLIMLTYLAPSIL-------HLN
CRE-SID-1       --------KKQKKMGSRKDAAK------FMIFFAVANFILMMMYLTSSKI-------HLN
CBR-SID-1       --------AKRKTLRSKQVYKV------FVGTFAIFNFFAITIYVTSSYI-------HLN
                           . :              .   :    :  :   :. : .       * :

CBR-CHUP-1      DFLSFLLIPFIGNLFIYIIYYILMKMIYREKIPKRAVALLIAAVISWTCAGILFNQRVSD
CBN-CHUP-1      DFLSFLLIPFIGNLFIYIIYYILMKMIHREKIPKRAMALLMAAVISWTCAGILFNQRVSD
CRE-CHUP-1      DFLSFLLIPFIGNLFIYIIYYILMKMIYREKIPKRALALLFAAVISWTCAGILFNQRVSD
CEL-CHUP-1      DFLSFLLIPFIGNLFIYIIYYILMKMIYREKIPKRAIALLFAAVISWTCAGILFNQRVSD
CJP-SID-1       QIVTYC---CIFNCIMYLIYYALIKYMCHERISHLAKLYGSLALVGWFVSAWFFFQDDMD
CEL-SID-1       QIATYC---FIINCIMYLMYYGCMKVLHSERITSKAKLCGALSLLAWAVAGFFFFQDDTD
CBN-SID-1       QIVTYC---FISNCIMYLVYYAVMKLVSRERIGLKGKVCGGLAVIGWIVAAVFFFQDDTD
CRE-SID-1       QITTYC---FILNCIVYLTYYATMKAVSRESIGLKAKICGTLAIVGWITAGYFFFQDDTD
CBR-SID-1       QISTYC---FILNCILYLTYYALMKFASRESIELKAKVCGVSAIFGWLIAGFFFFQDDTD
                :: ::     * * ::*: **  :*    * *   .      ::..*  :. :* *   *

CBR-CHUP-1      WSKMPAISRELNKPCIFLNFYDNHDIWHLSSAFAIFFSFTAINVIDDDLMFVMRNTIRVF
CBN-CHUP-1      WSKMPAISRELNKPCIFLNFYDNHDIWHLSSAFAIFFSFTAINVIDDDLMFVMRNTIKVF
CRE-CHUP-1      WSKMPAISRELNKPCIFLNFYDNHDIWHLSSAFAIFFSFTAINVIDDDLMFVVRNTIRVF
CEL-CHUP-1      WSKMPAISRELNKPCIFLNFYDNHDLWHLSSAFAIFFSFTAINVIDDDLMFVMRNTIRVF
CJP-SID-1       WTQSAAVSRSLNKPCVLLQFFGSHDLWHIFGAMAGLFTFLFVSYVDDDLLNIPKCSVTVF
CEL-SID-1       WTRSAAASRALNKPCLLLGFFGSHDLWHIFGALAGLFTFIFVSFVDDDLINTRKTSINIF
CBN-SID-1       WTRSSAASRALNKPCLLLNFFGSHDLWHIFGALAGLFTFLFVSFVDDDLVNTPKSAINVY
CRE-SID-1       WTRTAAASRALNKPCLLLDFFGSHDLWHIFGALAALFTFFSVSFVDDDLVNTPKSSINIF
CBR-SID-1       WTRTAAMSRALNTPCLLLDFFGSHDLWHMFGAIAGLFTFLFVSFVDDDLINTPKSSINMY
                *:. .* ** **.**::* *:..**:**: .*:* :*:*  :. :****:   . :: ::
                                       H740Y  G747R
```

Figure S1. A multiple sequence alignment of full-length SID-1 homologs. Available full-length SID-1 and CHUP-1 homologs from *C. elegans, C. brenneri, C. briggsae, C. remanei,* and *C. japonica* were aligned by MUSCLE v3.8 with default parameters (Edgar 2004). Residues identically conserved in all nine aligned sequences (predicted SID-1 orthologs and paralogs) are highlighted in black; residues identically conserved only in the five putative SID-1 orthologs are highlighted in grey. The position and amino acid change of the recovered weak alleles (regular weight) and strong alleles (bold weight) is indicated below the substituted residue.
