# Supplementary material for: SID-1 Domains Important for dsRNA Import in Caenorhabditis elegans
Source: G3 (Bethesda). 2017 Oct 12;7(12):3887–99. doi: 10.1534/g3.117.300308 (PMC5714486; doi:10.1534/g3.117.300308)
Supplement: Supplementary file 2 [file 3887FigureS2.pdf]

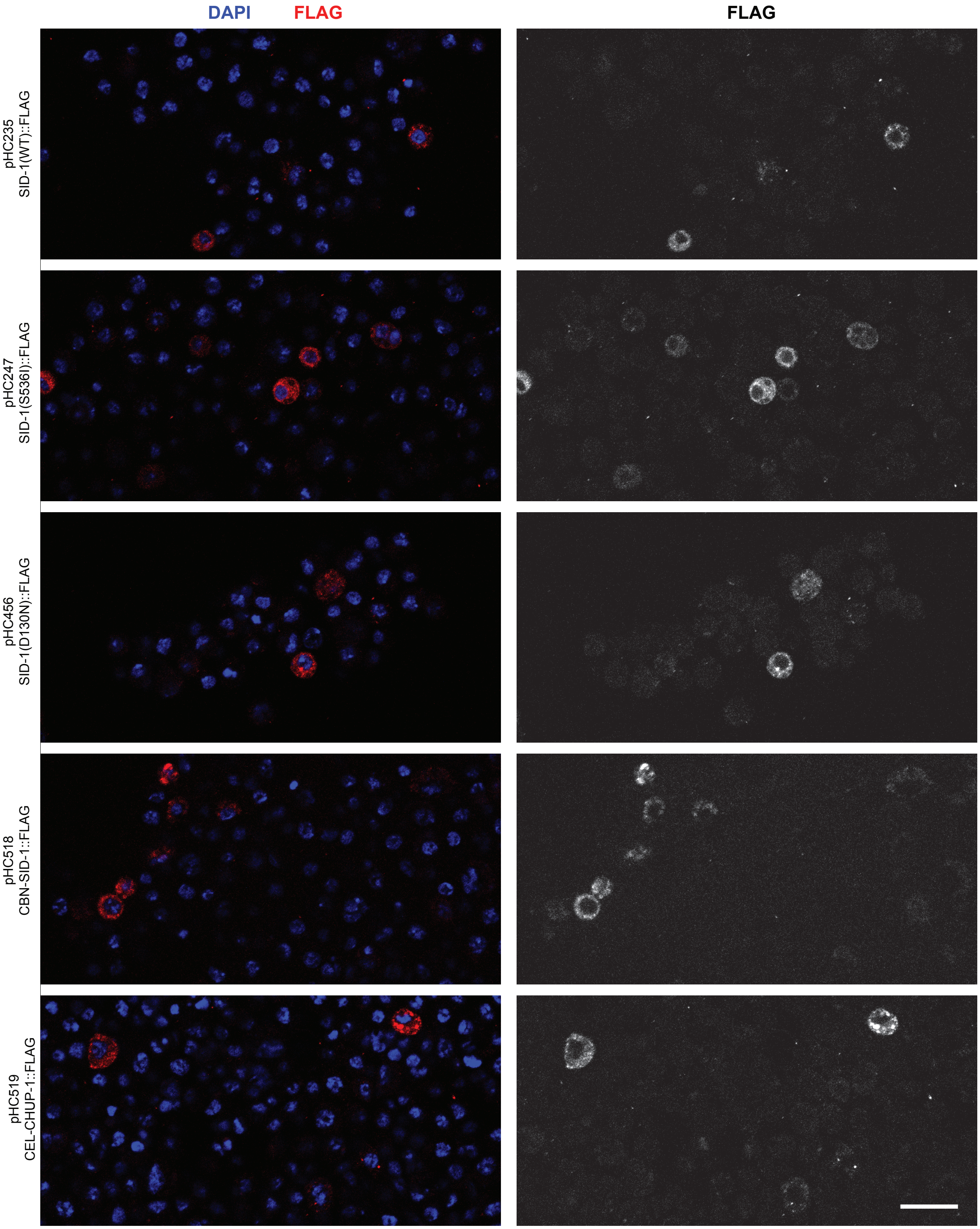

Figure S2. SID-1 homolog expression and localization in S2 cells. Anti-FLAG and DAPI staining (left column) and anti-FLAG staining only (right column) of Drosophila S2 cells transiently transfected with the indicated constructs. Scale bar 20 µm.
