# Supplementary material for: SID-1 Domains Important for dsRNA Import in Caenorhabditis elegans
Source: G3 (Bethesda). 2017 Oct 12;7(12):3887–99. doi: 10.1534/g3.117.300308 (PMC5714486; doi:10.1534/g3.117.300308)
Supplement: Supplementary file 3 [file 3887FileS1.pdf]

Table S1. Sequence analysis of *sid-1* alleles

Allele	Mutation location (domain)	DNA change	Amino acid change	RNAi phenotype†	Reference
qt6	N terminus	ATG-ATA	M1I	Weak	This study and (6)
qt84	N terminus	CCG-TCG	P108S	Strong	This study
*qt95, qt117, [pk3321]**	N terminus	GAT-AAT	D130N	Weak	This study and (15)
qt9, qt81	N terminus	CAG-TAG	Q154stop	Strong	This study and (6)
qt109	N terminus	CGG-TGG	R172W	Strong	This study
qt11, qt98, qt120*	N terminus	GCC-ACC	A173T	Weak	This study and (6)
qt100, qt110	N terminus	CAA-TAA	Q174stop	Strong	This study
qt10	N terminus	CCG-CTG	P199L	Weak	This study and (6)
qt97, qt104	N terminus	GGA-GAA	G201E	Weak	This study
qt113	N terminus	GGA-AGA	G201R	Strong	This study
qt86	N terminus	CAA-TAA	Q213stop	Strong	This study
qt5, qt107*	N terminus	CGA-TGA	R275stop	Strong	This study and (6)
qt91	N terminus	TAC-TAA	Y314stop	Strong	This study
qt7, qt8*, qt82*	TM1	CCG-CTG	P328L	Strong	This study and (6)
qt127	IC loop between TM1 and TM2	CAG-TAG	Q373stop	Strong	This study
qt92	IC loop between TM1 and TM2	CAA-TAA	Q402stop	Strong	This study
qt96	EC loop between TM2 and TM3	TGG-TGA	W452stop	Strong	This study
qt99, qt103	EC loop between TM2 and TM3	TGC-TAC	C464Y	Strong	This study
qt102	EC loop between TM2 and TM3	CGA-TGA	R478stop	Strong	This study
qt111	TM3	GGA-GAA	G488E	Strong	This study
qt80	TM3	GGA-AGA	G493R	Strong	This study
qt114	TM4	CAA-TAA	Q532stop	Strong	This study
qt85	TM4	ATT-AAT	I534N	Weak	This study
*qt2**	TM4	AGT-ATT	S536I		(6)
qt118	MR5	CAA-TAA	Q556stop	Strong	This study
qt112	MR5	TGT-TAT	C559Y	Strong	This study
qt4, qt93, qt115	EC loop between MR5 and TM6	CGT-TGT	R565C	Strong	This study and (6)
qt125	EC loop between MR5 and TM6	CGT-TGT	R570C	Strong	This study
qt87	IC loop between MR7 and MR8	CGA-TGA	R633stop	Strong	This study
qt90	EC loop between MR10 and MR11	CAC-TAC	H740Y	Strong	This study
qt101	EC loop between MR10 and MR11	TGG-TGA	W743stop	Strong	This study
qt130	MR11	GGA-AGA	G747R	Strong	This study
*qt12**	Splice donor site	G482A	Non-coding		(6)
qt129	Splice acceptor site	G524A	Non-coding	Strong	This study
qt89, qt108	Splice donor site	G5855A	Non-coding	Strong	This study
qt106	G to A, 24bp downstream of TAG	3'UTR	Non-coding	Strong	This study
qt78	385bp deletion from 3430-3815	Deletion		Strong	This study
qt83	338bp deletion from 5323-5661	Deletion		Strong	This study
qt88	465bp deletion from 2003-2468	Deletion		Strong	This study
qt116	158bp deletion from 5477-5635	Deletion		Strong	This study
qt119	378bp deletion from 6101-6479	Deletion		Strong	This study
qt126	Deletion from ~3600-4000	Deletion		Strong	This study
qt128	372bp deletion from 2005-2377	Deletion		Strong	This study

Table S2. Strong *sid-1* alleles on tissue-specific environmental RNAi

		act-5 RNAi gut		*bli-1 RNAi* hypodermis		*unc-22 RNAi* muscle		*unc-45 RNAi* muscle, germline			*fkh-6 RNAi* somatic gonad	
Mutation	Strain or Allele	% Arr	(n)	% Bli	(n)	% Unc	(n)	% Unc	Fertility	(n)	Fertility	(n)
CONTROLS												
	N2	100	(111)	100	(115)	100	(173)	100	Sterile	(237)	Sterile	(173)
	HC57	100	(64)	100	(34)	100	(43)	100	Sterile	(48)	Sterile	(12)
NONSENSE AND NON-CODING, STRONG												
Q154stop	*qt9*	0	(98)	0	(37)	0	(60)	0	Fertile	(24)	Fertile	(35)
Q154stop	*qt81*	0	(57)	0	(33)	0	(58)	0	Fertile	(68)	Fertile	(24)
Q174stop	*qt100*	0	(18)	6	(32)	0	(35)	0	Fertile	(24)	Fertile	(29)
Q174stop	*qt110*	0	(24)	0	(19)	0	(81)	0	Fertile	(74)	Fertile	(50)
Q213stop	*qt86*	0	(50)	ND		0	(31)	0	Fertile	(62)	Fertile	(57)
R275stop	*qt107*	0	(18)	0	(20)	0	(48)	0	Fertile	(42)	Fertile	(47)
Y314stop	*qt91*	0	(25)	ND		0	(51)	0	Fertile	(33)	Fertile	(38)
Q373stop	*qt127*	0	(23)	0	(17)	0	(19)	0	Fertile	(14)	Fertile	(17)
Q402stop	*qt92*	0	(16)	6	(33)	0	(17)	0	Fertile	(31)	Fertile	(33)
R478stop	*qt102*	0	(20)	0	(19)	0	(18)	0	Fertile	(22)	Fertile	(18)
W452stop	*qt96*	0	(35)	0	(34)	6	(35)	0	Fertile	(30)	Fertile	(35)
Q532stop	*qt114*	0	(27)	0	(22)	0	(22)	0	Fertile	(24)	Fertile	(18)
Q556stop	*qt118*	0	(23)	0	(9)	0	(15)	0	Fertile	(14)	Fertile	(25)
R633stop	*qt87*	0	(43)	ND		0	(44)	0	Fertile	(41)	Fertile	(35)
W743stop	*qt101*	0	(20)	0	(33)	0	(30)	0	Fertile	(27)	Fertile	(41)
Δ2003-2468	*qt88*	0	(65)	ND		0	(58)	0	Fertile	(50)	Fertile	(41)
Δ2005-2377	*qt128*	0	(14)	0	(18)	0	(23)	0	Fertile	(20)	Fertile	(16)
Δ3430-3815	*qt78*	0	(30)	0	(10)	0	(16)	4	Fertile	(23)	Fertile	(11)
~Δ3600-4000	*qt126*	0	(21)	0	(17)	0	(21)	0	Fertile	(14)	Fertile	(11)
Δ5323-5661	*qt83*	0	(57)	0	(53)	0	(67)	0	Fertile	(63)	Fertile	(32)
Δ5477-5635	*qt116*	0	(25)	0	(26)	0	(18)	0	Fertile	(14)	Fertile	(21)
Δ6101-6479	*qt119*	0	(22)	0	(25)	0	(44)	0	Fertile	(32)	Fertile	(29)
SAS G524A	*qt129*	0	(28)	0	(33)	0	(27)	0	Fertile	(30)	Fertile	(21)
SDS G5855A	*qt89*	0	(33)	ND		0	(43)	0	Fertile	(34)	Fertile	(35)
SDS G5855A	*qt108*	0	(18)	0	(12)	0	(56)	0	Fertile	(45)	Fertile	(28)
3'UTR G6649A	*qt106*	0	(12)	0	(14)	0	(39)	0	Fertile	(40)	Fertile	(13)
MISSENSE, STRONG												
P108S	*qt84*	0	(75)	0	(31)	0	(66)	0	Fertile	(60)	Fertile	(32)
R172W	*qt109*	0	(24)	0	(18)	0	(34)	0	Fertile	(56)	Fertile	(35)
G201R	*qt113*	0	(30)	0	(20)	0	(33)	0	Fertile	(14)	Fertile	(18)
P328L	*qt82*	0	(21)	0	(27)	0	(33)	0	Fertile	(28)	Fertile	(9)
C464Y	*qt99*	0	(20)	0	(36)	0	(36)	0	Fertile	(42)	Fertile	(33)
C464Y	*qt103*	0	(20)	0	(41)	3	(35)	0	Fertile	(38)	Fertile	(24)
G488E	*qt111*	0	(9)	0	(20)	0	(44)	0	Fertile	(51)	Fertile	(47)
G493R	*qt80*	0	(48)	0	(51)	0	(86)	0	Fertile	(52)	Fertile	(45)
C559Y	*qt112*	0	(28)	0	(23)	0	(50)	0	Fertile	(53)	Fertile	(48)
R565C	*qt93*	0	(28)	0	(20)	0	(24)	0	Fertile	(23)	Fertile	(16)
R565C	*qt115*	0	(36)	0	(37)	0	(24)	0	Fertile	(27)	Fertile	(31)
R570C	*qt125*	0	(38)	0	(15)	0	(28)	0	Fertile	(19)	Fertile	(16)
H740Y	*qt90*	0	(46)	ND		0	(45)	0	Fertile	(41)	Fertile	(46)
G747R	*qt130*	0	(18)	0	(29)	0	(33)	0	Fertile	(38)	Fertile	(24)

L1-L2 staged larvae were placed on bacteria expressing dsRNA at 20°C and were scored as young adults for the RNAi phenotypes. N2, wild type strain. HC57, background strain for all *sid-1* mutants. Arr, arrested; Bli, blistered cuticle; Unc, uncoordinated (*unc-45* RNAi animals are paralyzed, *unc-22* RNAi animals are twitching). Fertility was scored qualitatively: sterile (no progeny), fertile (many progeny), decreased (few progeny). (n), number of animals scored. ND, not determined.

Table S3. Sequences of primers used in this study.

A. For amplification and sequencing of the *sid-1* gene, C04F5.1

Primer	C04F5.1 starting nucleotide	Sequence
LGV W F	5' UTR	ggtcatgagagggtcgagag
C04F5.1 F1	5' UTR	tttttcccttcattttccatttt
C04F5.1 F2	283	gcggaaatcgttgattcttc
C04F5.1 F3	796	gcgaatgtacggattttgct
C04F5.1 F4	1352	cggaagaaaaattacctgattgg
C04F5.1 F5	1889	tcgatcagtgatttccgataaa
C04F5.1 F6	2263	gatttgagccatgatgagca
C04F5.1 F7	2719	cgaattgtcggagttggaat
C04F5.1 F8	4268	cgagcccagcgatacagtag
C04F5.1 F9	4750	tgggttgacgtaaaatgcaa
C04F5.1 F10	5297	ccccataaacatgaactgtcc
C04F5.1 F11	5781	ttgtggagctctgtcactgc
C04F5.1 R1	3' UTR	gcaaacgagcaattgtgaag
C04F5.1 R2	6526	gtgccataaatcgtgggaac
C04F5.1 R3	6000	cgggaaattaccgaaattga
C04F5.1 R4	5539	gatcgatccaacgacaatga
C04F5.1 R5	5021	aatcgcaataagacagcctga
C04F5.1 R6	4483	ggttgaaaattggcgaagaa
C04F5.1 R7	3062	tgaaacggccgacttttact
C04F5.1 R8	2471	tcaagccaaatatccgcaag
C04F5.1 R9	2012	cgggagctatgaagacgaag
C04F5.1 R10	1550	ttttggaaaatcaagcaaaaa
C04F5.1 R11	1004	tgatcgattcaaacaaaattcg
C04F5.1 R12	500	tttaggcccaaaagcctacc
C04F5.1 new genomic F	3769	cacaatcatgcgtgtgctc
C04F5.1 new genomic R	3877	tcatgacggcctcttctaca
sid-1 Exon 5 F	3473	cgcgccatccatagatttta
sid-1 Exon 5 R	4002	catcactggggcaaatatga
sid-1 Exon 3 R	827	ttgaaatttaaagcaaaatccgta

B. For amplification of the *cbn-sid-1* promoter, gene (CBN00847) and 3' UTR

Primer	Sequence
AW179_cbn-sid-1 genomic_F1	tggcatgagttctgaaatgaa
AW180_cbn-sid-1 genomic_R1	tgttcaagttcaagtggcaag

C. For cloning *sid-1* homolog cDNAs onto pPacPL and site-directed mutagenesis of the *sid-1* cDNA sequence on pHC235.

Primer	Sequence
AW168_cbn-sid-1 cDNA+overlap_F	tagactcgagccgcaatgaaaggcagcggctcc
AW169_cbn-sid-1 cDNA+overlap_R	cttgtcgtcgtcgtccttgtagtcatacacgttaattgcggattttg
AW170_pPacPL backbone plus flag_F	gactacaaggacgacgacg

AW171_pPacPL backbone plus flag_F	tgcggctcgagtctagag
AW205_tag-130 cDNA+overlap_F	tagactcgagccgcaatgaggacctcacaggcg
AW206_tag-130 cDNA+overlap_R	cttgtcgtcgtcgtccttgtagtcgaaaactcgaattgtattcctcatc
3428 qt95 Forward (D130N substitution)	caatccgctcgttgagcaaaatttcggggcgcacagaaag
3429 qt95 Reverse (D130N substitution)	ctttctgtgcgccccgaaattttgctcaacgagcggattg

D. For gut and muscle rescue of *sid-1*

Primer	Sequence
P5	catttggcgaggagccatg
P23	gtacgacagaaagcacttcg
P37	gcggcatggttttggtaac
P38	cagttcaagacagtttggtg

Table S4. Strains used in this study

Strain	Genotype
N2 Bristol	wild type
HC195	*nrIs20 [sur-5::NLS-gfp] IV*
HC196	*sid-1(qt9) V*
HC393	*sid-1(qt9) V; qtEx89 [sur-5::NLS-gfp + sid-1(+)]*
HC566	*nrIs20 [sur-5::NLS-gfp] IV; sid-1(qt9) V*
HC757	*nrIs20 [sur-5::NLS-gfp] IV; sid-1(qt95) V*
HC57	*ccIs4251[myo-3::NLS-gfp, myo-3::MITO-gfp] I; qtIs3 [myo-2::GFP dsRNA] III; mIs11 [myo-2::GFP] IV*
HC303	*sid-1(qt6) V*
HC94	*sid-1(qt10) V*
HC653	*sid-1(qt78) V*
HC655	*sid-1(qt80) V*
HC656	*sid-1(qt81) V*
HC657	*sid-1(qt82) V*
HC658	*sid-1(qt83) V*
HC659	*sid-1(qt84) V*
HC660	*sid-1(qt85) V*
HC661	*sid-1(qt86) V*
HC662	*sid-1(qt87) V*
HC663	*sid-1(qt88) V*
HC664	*sid-1(qt89) V*
HC665	*sid-1(qt90) V*
HC666	*sid-1(qt91) V*
HC667	*sid-1(qt92) V*
HC668	*sid-1(qt93) V*
HC670	*sid-1(qt95) V*
HC671	*sid-1(qt96) V*
HC672	*sid-1(qt97) V*
HC673	*sid-1(qt98) V*
HC674	*sid-1(qt99) V*
HC675	*sid-1(qt100) V*
HC676	*sid-1(qt101) V*
HC677	*sid-1(qt102) V*
HC678	*sid-1(qt103) V*
HC679	*sid-1(qt104) V*
HC681	*sid-1(qt106) V*
HC682	*sid-1(qt107) V*
HC683	*sid-1(qt108) V*
HC684	*sid-1(qt109) V*
HC685	*sid-1(qt110) V*
HC686	*sid-1(qt111) V*
HC687	*sid-1(qt112) V*
HC688	*sid-1(qt113) V*
HC689	*sid-1(qt114) V*
HC690	*sid-1(qt115) V*

HC691	*sid-1(qt116) V*
HC692	*sid-1(qt117) V*
HC693	*sid-1(qt118) V*
HC694	*sid-1(qt119) V*
HC709	*sid-1(qt120) V*
HC714	*sid-1(qt125) V*
HC715	*sid-1(qt126) V*
HC716	*sid-1(qt127) V*
HC717	*sid-1(qt128) V*
HC718	*sid-1(qt129) V*
HC719	*sid-1(qt130) V*
HC935	*sid-1(qt95) V; qtEx157[sid-2::sid-1 cDNA, myo-3::dsRed]*
HC936	*sid-1(qt95) V; qtEx157[sid-2::sid-1 cDNA, myo-3::dsRed]*
HC937	*sid-1(qt95) V; qtEx158[myo-3::sid-1 cDNA, myo-3::dsRed]*
HC938	*sid-1(qt95) V; qtEx158[myo-3::sid-1 cDNA, myo-3::dsRed]*
HC1034	*sid-1(qt9) V; qtEx195[cbn-sid-1 promoter::cbn-sid-1::3'UTR; pHC183 myo-3::dsRed]*
PB2801	*Caenorhabditis brenneri*, outcrossed x20

Strains HC94 and HC653 through HC719 are in the HC57 strain background.
